# Engineered *Pseudomonas putida* KT2440 co-utilizes galactose and glucose

**DOI:** 10.1186/s13068-019-1627-0

**Published:** 2019-12-23

**Authors:** George L. Peabody, Joshua R. Elmore, Jessica Martinez-Baird, Adam M. Guss

**Affiliations:** 1 Biosciences Division, Oak Ridge National Laboratory, Oak Ridge, TN 37831 USA; 2 Present Address: Biological Sciences Division, Pacific Northwest National Laboratory, Richland, WA 99354 USA

**Keywords:** Galactose, Co-utilization, De Ley–Doudoroff, *Pseudomonas putida* KT2440

## Abstract

**Background:**

Efficient conversion of plant biomass to commodity chemicals is an important challenge that needs to be solved to enable a sustainable bioeconomy. Deconstruction of biomass to sugars and lignin yields a wide variety of low molecular weight carbon substrates that need to be funneled to product. *Pseudomonas putida* KT2440 has emerged as a potential platform for bioconversion of lignin and the other components of plant biomass. However, *P. putida* is unable to natively utilize several of the common sugars in hydrolysate streams, including galactose.

**Results:**

In this work, we integrated a De Ley–Doudoroff catabolic pathway for galactose catabolism into the chromosome of *P. putida* KT2440, using genes from several different organisms. We found that the galactonate catabolic pathway alone (DgoKAD) supported slow growth of *P. putida* on galactose. Further integration of genes to convert galactose to galactonate and to optimize the transporter expression level resulted in a growth rate of 0.371 h^−1^. Additionally, the best-performing strain was demonstrated to co-utilize galactose with glucose.

**Conclusions:**

We have engineered *P. putida* to catabolize galactose, which will allow future engineered strains to convert more plant biomass carbon to products of interest. Further, by demonstrating co-utilization of glucose and galactose, continuous bioconversion processes for mixed sugar streams are now possible.

## Background

The vast majority of global fuels and platform chemicals are produced from petroleum. However, petroleum is a finite resource, so synthesizing platform chemicals from renewable feedstocks is needed for a sustainable future. Biological valorization of sugars and lignin from plant-based biomass to commodity chemicals is a potential route to renewable and sustainable alternatives. Although different feedstocks and pretreatment processes yield different available substrates for microbial conversion, several sugars are regularly detected in hydrolysate streams [1, 2]. While glucose is typically the most abundant, xylose, galactose, mannose, and arabinose are all present as well at different concentrations. In order to effectively convert these sugars to product, an ideal organism would at a minimum require high tolerance to inhibitors and rapid sugar catabolism.

*Pseudomonas putida* KT2440 is emerging as a new favorite synthetic biology chassis for biocatalysis of deconstructed biomass [3]. *P. putida* KT2440 has a wide range of genetic tools available [4–7], a well-characterized metabolism well suited for redox-intensive transformations [8–11], demonstrated ability to host a variety of heterologous pathways in vivo for a vastly enlarged biochemical work space [12, 13], and established scale-up capabilities. For example, *P. putida* KT2440 has been engineered to grow anoxically [14], to catabolize novel substrates [15–17], and to synthesize a diverse array of chemicals [3, 12]. Moreover, *P. putida* KT2440 has been successfully engineered to utilize both of the common hemicellulosic pentoses: xylose and arabinose [18–20]. However, *P. putida* KT2440 has not been engineered to catabolize galactose, the next most abundant sugar in many hemicelluloses, which can be up to 3% of total sugars in plant biomass [21]. It will be important to capture this carbon for an efficient bioconversion process.

**Fig. 1:**
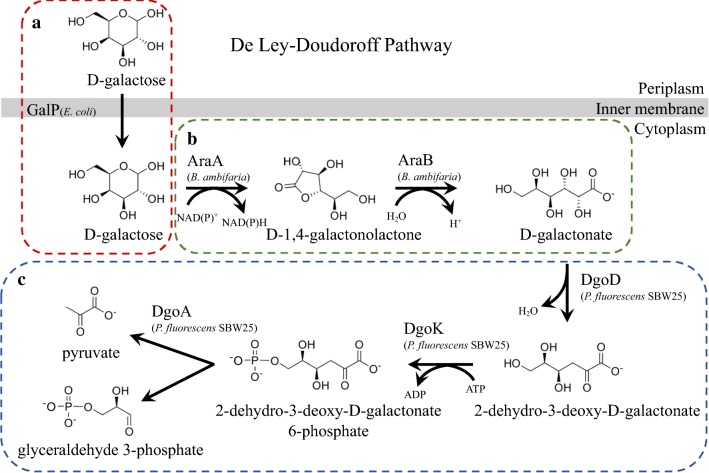
Components of the DLD galactose pathway used in this study. **a** Transport across the inner membrane, outlined in red. **b** Galactose conversion to galactonate, outlined in green. **c** Galactonate conversion to pyruvate and glyceraldehyde 3-phosphate, outlined in blue. *GalP* galactose–proton symporter, *DgoK* 2-dehydro-3-deoxygalactonokinase, *DgoA* 2-dehydro-3-deoxy-6-phosphogalactonate aldolase, *DgoD* d-galactonate dehydratase, *AraA* l-arabinose 1-dehydrogenase/d-galactose dehydrogenase, *AraB*, l-arabinolactonase/d-galactonolactonase

There are two common pathways for galactose catabolism in bacteria: the Leloir (LL) pathway and the De Ley–Doudoroff (DLD) pathway [2]. In the LL pathway, galactose is phosphorylated and then converted to glucose-1-phosphate through a cyclic pair of transferase/epimerase reactions with uridyl monophosphate intermediates [22, 23]. The DLD pathway, on the other hand, mirrors the Entner–Doudoroff (ED) pathway for glucose catabolism used by *P. putida* KT2440, wherein the sugar is ultimately converted to glyceraldehyde 3-phosphate (G3P) and pyruvate (PYR) [24]. The DLD pathway can be separated into three parts: transport (Fig. 1a), galactose conversion to galactonate (Fig. 1b), and galactonate to G3P and PYR (Fig. 1c). Transport of galactose into the cell is relatively well studied with numerous sugar transporters reported to have activity on galactose as either a primary or secondary substrate; for example, GalP from *Escherichia coli* is a sugar-proton symporter of both galactose and glucose [25]. The second portion of the DLD pathway, where galactose is converted to galactonate, is less well characterized. Some organisms have been described to have these activities. Although enzymes with dehydrogenase and lactonase activity on galactose and 1,4-galactonolactone have been identified, such as AraAB from *Burkholderia ambifaria*, no sequence of a specific galactonolactonase has been identified [26–30]. For the last portion of the DLD pathway, three enzymatic steps of dehydration, phosphorylation, and subsequent aldol cleavage are performed by DgoD, DgoK, and DgoA, respectively (Fig. 1c) [31]. Homologs of these proteins are encoded in a wide variety of organisms, including many pseudomonads such as *Pseudomonas fluorescens* SBW25. Interestingly, these genes are even encoded in some organisms that use the LL pathway like *E. coli*, where the *dgoKAD* operon is a separately regulated pathway only for galactonate catabolism. However, to the best of our knowledge, the complete DLD pathway has not been successfully introduced into an organism that does not natively utilize galactose and allowed for growth with galactose as the sole carbon source.

To expand the substrate range of *P. putida* KT2440 to include galactose, in this study, we harness the less commonly used DLD pathway of galactose catabolism. We chose this pathway because it is observed in pseudomonads, and the products of the DLD pathway (G3P and PYR) are the same as the ED pathway natively used by *P. putida* KT2440 for glucose catabolism. Additionally, the DLD pathway catabolizes galactose via different metabolic intermediates relative to glucose, whereas the Leloir pathway uses the exact same intermediates and may compete for the same flux space. Therefore, the DLD pathway might lead to better sugar co-utilization. Here we built a functional DLD pathway using genes from *E. coli*, *P. fluorescens* SBW25, and *B. ambifaria*. We then demonstrated the ability of this strain to utilize galactose alone and co-utilize galactose and glucose.

## Results and discussion

### Heterologous expression of DgoKAD allows growth on galactose

**Fig. 2:**
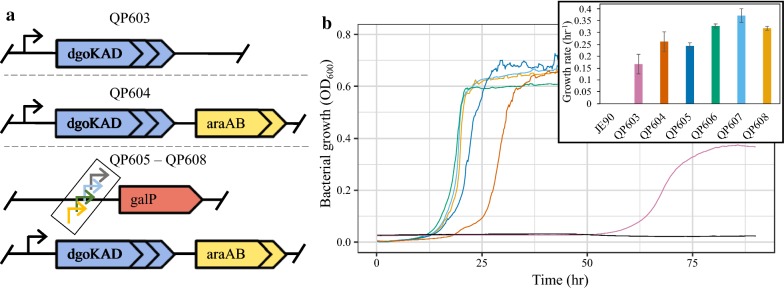
Schematic of strains constructed and their growth on galactose. **a** Pictorial representation of genotypes for strains QP603–QP608 (see “Materials and methods” for additional details). **b** Growth of each strain on MME media with 10 mM galactose as the sole carbon source. Strains JE90 (parent strain), black; QP603 (JE90::*dgoKAD*), pink; QP604 (QP603::*araAB*), dark orange; QP605 (QP604::*P*_*1548*_-*galP*), dark blue; QP606 (QP604::*P*_*3079*_-*galP*), green; QP607 (QP604::*P*_*lac*_-*galP*), light blue; QP608 (QP604::*P*_*tac*_-*galP*), yellow. The inset shows the measured growth rate for each strain. Strains were grown at 30 °C aerobically in a 48-well microtiter plate in biological triplicate with measurements every 10 min

Wild-type *P. putida* KT2440 does not grow on galactose or galactonate, so we first explored what portions of the DLD pathway were required for galactose catabolism [32]. Glucose dehydrogenases in pseudomonads can have a wide substrate range, and there are many uncharacterized and promiscuous sugar transporters in *P. putida* KT2440 [18]. We therefore hypothesized that side activity of native enzymes may be sufficient for galactose transport and conversion to galactonate. Therefore, we introduced a galactonate conversion pathway into the chromosome of *P. putida* by expressing the *dgoKAD* operon from *P. fluorescens* SBW25 under its native promoter, creating strain QP603 (Fig. 2a, Table 1). We inoculated strain QP603 into minimal medium with galactose as the sole carbon and energy source and observed slow growth on galactose after a 52-h lag phase (Fig. 2b, Additional file 1: Table S1). No accumulation of galactonate or any other molecule was seen in the supernatant by HPLC. This demonstrates that expression of *dgoKAD* is sufficient for galactose catabolism and that native systems must be capable of galactose transport and oxidation to galactonate at a low level.

**Table 1:** Strains and plasmids used in this study

Strains	Genotype/plasmid	Source
JE90	*P. putida* KT2440 ∆PP_4740::P_tac_BXB1int-*attB*_BxB1_	[17]
QP603	JE90 ∆PP_0545::*dgoKAD*	This work
QP604	JE90 ∆PP_0545::*dgoKAD*:*araAB*	This work
QP605	QP604 *attB*_BxB1_::pQP344	This work
QP606	QP604 *attB*_BxB1_::pQP345	This work
QP607	QP604 *attB*_BxB1_::pQP346	This work
QP608	QP604 *attB*_BxB1_::pQP347	This work
*E. coli* F’I^Q^	*E. coli* F’I^Q^	NEB
Plasmids		
pK18mobsacB	pUC origin, KanR, origin of transfer, *sacB* counter selectable marker	[18]
pJE1045	“Cargo” plasmid for chromosomal integration, BxB1 *attP*, pUC origin, KanR, and P_tac_:mNeongreen	[17]
pJE1553	pk18mobsacB based, for ∆PP_0545::*dgoKAD* mutation	This work
pQP348	pk18mobsacB based, for ∆PP_0545::*dgoKAD*:*araAB* mutation	This work
pQP344	pJE1045 with P_PP_1548_:*galP*_ppopt_	This work
pQP345	pJE1045 with P_PP_3079_:*galP*_ppopt_	This work
pQP346	pJE1045 with P_lac_:*galP*_ppopt_	This work
pQP347	pJE1045 with P_tac_:*galP*_ppopt_	This work

### Growth rate improved by pathway expansion

We next examined improving the growth rate of strain QP603. While the side activity of native enzymes could perform the transport, dehydrogenase, and lactonase activities of the DLD pathway, they could be rate limiting. We therefore introduced enzymes to convert intracellular galactose to galactonate. Because no galactose-specific dehydrogenase and lactonase are yet to be identified, we assembled our pathway using the best-characterized galactonolactonase and its associated galactose dehydrogenase that are currently known—an arabinose dehydrogenase, AraA, and arabinonolactonase, AraB, from the oxidative arabinose pathway of *B. ambifaria* [26, 27, 30]. We introduced codon-optimized versions of *araA* and *araB* into strain QP603 at the end of the *dgoKAD* operon, resulting in a longer operon of five genes *dgoKAD:araAB* and a new strain QP604 (Fig. 2a). This strain grew 60% faster than strain QP603 and had a substantially reduced lag phase (Fig. 2b, Additional file 1: Table S1). Unfortunately, strain QP604 still experienced a significant lag of approximately 26 h, and the growth rate was still slower than that of glucose catabolism despite having a similar energetic yield and producing the same central metabolites, G3P and PYR.

Because we had introduced all the catabolic parts of the DLD pathway, we hypothesized that growth on galactose may now be limited by substrate uptake. We selected the *E. coli* GalP to study the impact of transport on growth rate in strain QP604. A codon-optimized *galP* was introduced into the BxB1 *attB* site of strain QP604 using site-specific recombination with four different promoters to generate strains QP605–QP608 [6]. Promoters of increasing strength were used to express *galP*, including the upstream regions of PP_1548 and PP_3079 and the *E. coli lac* and *tac* promoters (Additional file 1: Table S2), resulting in strains QP605 to QP608, respectively. All four strains showed a significantly reduced duration of lag phase (*P* value < 0.001) and the growth improved in strains QP606–QP608 (*P* value < 0.01) when compared to the parent QP604 for growth on galactose (Fig. 2b, Additional file 1: Table S1). Expression of *galP* with the *lac* promoter in strain QP607 had the greatest increase in growth rate relative to QP604, 41%, with an overall growth rate of 0.371 ± 0.03 h^−1^. This expression optimization for the galactose transporter suggests that growth rate improves with higher *galP* expression up to the strength of the *lac* promoter. However, growth rate decreased when using the very highly expressed *tac* promoter, suggesting that overexpression of this transporter can become toxic to our engineered cells. Comparatively, the WT grown under identical conditions but with glucose as the carbon source grew at 0.87 ± 0.09 h^−1^, about 2.5-fold faster. Overall, while the introduction of *dgoKAD* was sufficient to supply growth on galactose, the complete DLD pathway including a transporter was required for rapid catabolism of galactose as the sole carbon source.

### Galactose is co-utilized with glucose

**Fig. 3:**
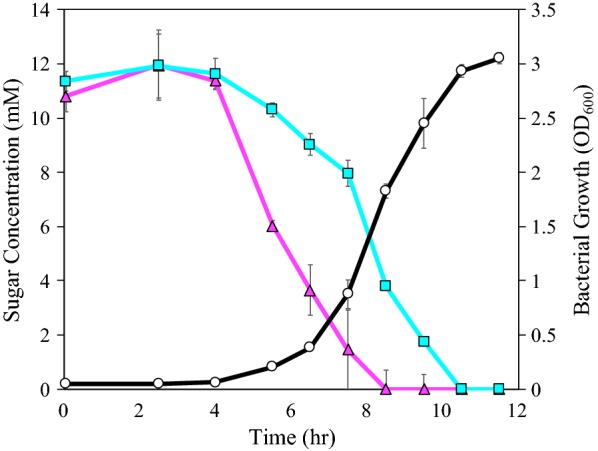
Shake flask characterization of strain QP607 growth on glucose and galactose. HPLC data for glucose and galactose concentrations are shown in magenta triangles, and cyan squares, respectively. Bacterial growth, as measured by a change in optical density, is shown in black circles

Co-utilization allows for faster and potentially continuous approaches to bioprocessing, making it important for future commercialization. We therefore sought to determine whether galactose could be co-utilized with glucose. To test whether co-utilization indeed occurs, we measured sugar utilization of strain QP607 in shake flasks with equimolar amounts of glucose and galactose. Both glucose and galactose were simultaneously utilized, no additional peaks such as for galactonate accumulation were observed in the supernatant quantification, and the growth of strain QP607 did not have a diauxic shape (Fig. 3). Together, this evidence demonstrates co-utilization of glucose and galactose in strain QP607. It is not surprising strain QP607 was able to co-utilize galactose and glucose simultaneously. *P. putida* does not natively utilize galactose, so the heterologous DLD pathway should be unregulated in *P. putida*. Furthermore, the pathway produces the same products as the natively utilized ED pathway for glucose catabolism, so it was expected the DLD pathway would be able to seamlessly integrate into central metabolism when the strain is growing on glucose. However, we did observe that glucose was utilized more rapidly than galactose in QP607. Fortunately, because galactose is a less abundant sugar than glucose in lignocellulose (approximately 1:30 ratio in corn stover hydrolysate [2]), the current slower utilization rate of galactose should still be sufficient for most real-world settings. Based on the similarity to the ED pathway and the lack of detected products, the galactose was presumably completely oxidized to CO_2_ via the TCA cycle in these strains.

While the current level of pathway functionality is likely sufficient for most applications, additional research could lead to faster galactose catabolism. In this work, we primarily focused on tuning expression of the transporter because although substrate uptake is critical, membrane protein overexpression can be toxic. Similar tuning of the rest of the genes in the pathway could further improve the growth rate. Other approaches such as adaptive laboratory evolution would also likely result in more rapid galactose catabolism. Finally, metabolomics studies could help reveal how the newly introduced DLD pathway integrates with the cyclic EDEMP pathway, which may be critical for future metabolic engineering efforts to divert flux away from growth and toward product formation.

## Conclusions

We have expanded the potential for total hydrolysate biocatalysis by *P. putida* KT2440, an emerging model organism for synthetic biology and biomass valorization, by introducing the DLD pathway for galactose catabolism. In doing so, we have determined the enzymes required for rapid catabolism and optimized the expression level of galactose transport. Furthermore, we have shown that this pathway allows co-consumption of galactose with a preferred substrate such as glucose. This work not only further demonstrates the strength of *P. putida* as a modular biocatalysis chassis, but also benefits the community developing bioprocesses for total hydrolysate conversion by expanding the catabolic sugar profile of *P. putida*. We plan to expand this work in the future by incorporating the previously demonstrated catabolic pathways for xylose and arabinose into our galactose utilizing strain.

## Materials and methods

### Strain construction

*Pseudomonas putida* strain JE90 (*P. putida* KT2440 *∆hsdR::BxB1int*-*attB* [6]) was the parent for all strains made in this study. For the plasmids to insert *dgoKAD* and *araAB* into the chromosome, the genes were cloned into pK18mobsacB [33] flanked by 1 kb sequences identical to the upstream and downstream region of PP_0545, a non-specific aldehyde dehydrogenase. These regions are used for homologous recombination to replace PP_0545 with the pathway genes. Primers were from (Eurofins Genomics, Louisville, KY), and gBlocks for codon-optimized *araAB* from *B*. *ambifaria* ATCC BAA-244 and *galP* from *E*. *coli* were synthesized by Integrated DNA Technologies. Genomic DNA was used to amplify *dgoKAD*, and its native promoter from *P. fluorescens* SBW25 and gDNA from *P. putida* strain JE90 was amplified using Phusion High Fidelity Polymerase (Thermo Fisher Scientific, Waltham, MA) when relevant. Plasmids were constructed via Gibson assembly with the NEBuilder HiFi DNA assembly master mix (New England Biolabs (NEB), Ipswich, MA), DNA was extracted from agarose gels with Zymo gel extraction kit (Zymo Research, Irvine, CA) and transformed into NEB F’I^Q^ *E. coli* chemically competent cells following the manufacturers guidelines (NEB). Plasmid sequences were confirmed via sequencing by Eurofins Genomics, and annotated plasmid sequences are available in Additional files 2, 3, 4, 5, 6, and 7. Strain genotypes and plasmids used in this study are listed in Table 1. When necessary, all antibiotic selections were performed with 50 µg/mL kanamycin. Plasmids were harvested with the geneJET miniprep kit (Thermo Fisher Scientific). For seamless chromosomal editing to introduce *dgoKAD* and *araAB* cassettes into *P. putida,* pK18mobsacB-derived plasmids were transformed into *P. putida* and mutants were selected using kanamycin selection and sucrose counter-selection as previously described [34]. The promoter–transporter cassettes were integrated using BxB1 phage integration as previously described [6].

### Growth medium

Utilization of galactose and glucose was tested aerobically in the MOPS-buffered minimal medium MME, which consisted of (per liter): 1.6 g K_2_HPO_4_·3 H_2_O, 4.2 g 3-(*N*-morpholino)propanesulfonic acid (MOPS), 0.25 g NaCl, 0.50 g NH_4_Cl, 0.10 g MgSO_4_·7 H_2_0, 0.01 g CaCl_2_·2 H_2_O, and 1 mL of a 1000× trace element solution, which consisted of (per liter): 1.00 mL concentrated HCl, 0.50 g Na_4_EDTA·H_2_O, 2 g FeCl_3_, 0.05 g H_3_BO_3_, 0.05 g ZnCl_2_, 0.03 g CuCl_2_·2H_2_O, 0.05 g MnCl_2_·4H_2_O, 0.05 g (NH_4_)2MoO_4_, 0.05 g CoCl_2_·6H_2_O, and 0.05 g NiCl_2_·6H_2_O. Glucose and galactose were added as growth substrates at the concentrations detailed below.

### Plate reader growth assays

Strains were grown from single colonies in LB overnight at 30 °C with shaking at 250 rpm. Cells were washed in substrate-free MME medium and a 1% inoculum was transferred to MME medium supplemented with 10 mM galactose. After the cells had reached stationary phase, 10 μL of each sample were further passaged into 500 μL MME medium supplemented with 10 mM galactose in a 48-well plate (Greiner Bio-One). Edge wells of the plate were filled with 700 μL media and not used for data collection to minimize the impact of evaporation. Data were collected on an Epoch2 plate reader (BioTek, Winooski, VT) with fast continuous double orbital shaking at 30 °C aerobically. A temperature gradient of 1 °C was added to minimize condensation. Measurement of OD_600_ was performed every 10 min. Growth rates were calculated with CurveFitter software [35] using only linear regions of growth on a log(OD_600_) vs time plot, and only OD_600_ values below 25% OD_600_ max. The lag phase was also calculated with the CurveFitter software. *P* values were calculated with Student’s *t* test.

### Shake flask growth assay

Strain QP607 was grown to mid-log phase in MME medium supplemented with 12 mM glucose and 12 mM galactose. The cells were transferred to a 125-mL flask containing 25 mL of the glucose–galactose MME medium at 30 °C with 250-rpm shaking. The cells were monitored for growth, and periodically 1 mL was sampled to measure OD_600_ and for HPLC analysis.

### Galactose and glucose HPLC quantification

Samples were filtered with 0.2-μm Corning Costar Spin-X centrifuge tube filters and then acidified with H_2_SO_4_ to a final concentration of 5 mM. The samples were run on a Waters HPLC equipped with refractive index detector and a Supelcogel H 6% column with a 0.6 mL/min flow rate of 5 mM H_2_SO_4_ in water as the running buffer at 60 °C. Sugar concentrations were determined by comparison to a standard curve.

## Supplementary information

**Additional file 1.** Supplemental Methods and Tables.

**Additional file 2.** Plasmid map for pJE1553.

**Additional file 3.** Plasmid map for pQP348.

**Additional file 4.** Plasmid map for pQP344.

**Additional file 5.** Plasmid map for pQP345.

**Additional file 6.** Plasmid map for pQP346.

**Additional file 7.** Plasmid map for pQP347.

## Data Availability

All data generated or analyzed during this study are included in this published article and its additional information files.

## Abbreviations

LL pathway: Leloir pathway
DLD pathway: De Ley–Doudoroff pathway
ED pathway: Entner–Doudoroff pathway
G3P: glyceraldehyde-3-phosphate
PYR: pyruvate
