# Supplementary material for: Engineered Pseudomonas putida KT2440 co-utilizes galactose and glucose
Source: Biotechnol Biofuels. 2019 Dec 23;12:295. doi: 10.1186/s13068-019-1627-0 (PMC6927180; doi:10.1186/s13068-019-1627-0)
Supplement: Supplementary file 1 — Additional file 1. Supplemental Methods and Tables. [file 13068_2019_1627_MOESM1_ESM.docx]

Additional Materials and Methods:

MME media recipe:

Per Liter of standard MME:

1.6 g K2HPO4 – 3 H2O (~9.1 mM)

4.2 g MOPS (~20 mM)

0.25 g NaCl (4.3 mM)

0.50 g NH4Cl (9.3 mM)

0.10 g MgSO4 – 7 H20 (0.41 mM)

0.01 g CaCl2 – 2 H2O (68 uM)

1 mL 1000x MME trace minerals

pH to 7.0 with KOH and bring to 1 L volume

Sterilize by filter sterilization before use (add sterile carbon source after MME sterilization).

**1000X MME Trace minerals**

Ingredient 1000 mL MW (g/mol) [Final] mM

HCl (concentrated) 1.00 mL --- ---

Na_4_EDTA⋅xH_2_O (tetrasodium) 0.50 g 380.17 1.31

FeCl_3_ 2 g 162.2 12.2

H_3_BO_3_ 0.05 g 61.83 0.808

ZnCl_2_ 0.05 g 136.315 0.367

CuCl_2_⋅2H_2_O 0.03 g 170.48 0.176

MnCl_2_⋅4H_2_O 0.05 g 197.91 0.253

(NH_4_)_2_MoO_4_ 0.05 g 196.01 0.255

CoCl_2_⋅6H_2_O 0.05 g 237.93 0.210

NiCl_2_⋅6H_2_O 0.05 g 237.69 0.210

dH_2_O to 1 L

Add HCl and EDTA to ~900 mL dH_2_O. Then dissolve remaining salts and fill volume to 1000 mL. Solution is clear yellow. Store at room temperature (do not autoclave). Filter sterilize, if desired. *Note: Do not place your pipette directly into a glass 2.5 L HCl bottle, transfer a small volume into a beaker or glass stoppered bottle and dispense from there.*

Promoter strength measurement methods:

Four plasmids were constructed to test promoter strength. Plasmids pQP344-347 were each digested with NdeI and XbaI and ligated to a similarly digested fragment of the *mkate*2 gene. Each plasmid was sequence-verified and then transformed into JE90 as outlined in the Materials and Methods. Three single colony isolates of each transformation were selected and grown up on LB media supplemented with kanamycin 50 ug/mL. The cultures were spun down at 3000x g and resuspended in MME media with no carbon source. 5 µl of each washed culture were inoculated into separate wells of a skirted, glass bottom, black, 96 well plate (Greiner Bio-One, Monroe, NC). Each of the central wells of the plate reader were filled with 100 µl of MME media with 10 mM glucose. The edge wells were filled with 150 µl media. The fluorescence plate reader, NEO2 (BioTek, Winooski, VT), was set to measure absorbance and fluorescence every ten minutes. Absorbance was measured at 600 nm and fluorescent emission was measured with an excitation wavelength of 568 nm and measurement at 588 nm. Additionally, the plate reader was set to incubate at 30°C with double orbital fast shaking; to minimize evaporation, no edge wells were utilized and a temperature gradient of 2 ºC was set. Data is in Additional Table S2.

Additional Table S1: Lag phase data and calculations.

| Calculations of Lag | | | | | | |
| --- | --- | --- | --- | --- | --- | --- |
| Strain | QP603 | QP604 | QP605 | QP606 | QP607 | QP608 |
| Experimental run 1 | 46.8 | 26.0 | 15.8 | 15.5 | 16.1 | 16.4 |
| Experimental run 2 | 46.1 | 26.1 | 16.0 | 15.7 | 16.5 | 16.0 |
| Experimental run 3 | 64.2 | 26.4 | 15.4 | 15.8 | 16.1 | 16.2 |
| Average lag | 52.4 | 26.2 | 15.7 | 15.7 | 16.2 | 16.2 |
| Standard deviation of lag | 10.0 | 0.2 | 0.3 | 0.2 | 0.2 | 0.2 |
| P-value vs QP603 | N/A | 0.047 | 0.025 | 0.025 | 0.026 | 0.026 |
| P-value vs QP604 | 0.025 | N/A | 4.0E-06 | 7.0E-07 | 7.2E-07 | 4.8E-07 |

Additional Table S2: Promoter activity as measured by mKate fluorescence, reported in relative fluorescence units (RFU)/optical density (OD_600_).

| Promoter | Strength (RFU/OD600) | Standard deviation |
| --- | --- | --- |
| *P_1548* | 56.9 | 13.1 |
| *P_3079* | 2050 | 128 |
| *Plac* | 3460 | 116 |
| *Ptac* | 12600 | 440 |
